# The oldest unvaccinated Covid-19 survivors in South America

**DOI:** 10.1186/s12979-022-00310-y

**Published:** 2022-11-16

**Authors:** Mateus V. de Castro, Monize V. R. Silva, Michel S. Naslavsky, Marilia O. Scliar, Kelly Nunes, Maria Rita Passos-Bueno, Erick C. Castelli, Jhosiene Y. Magawa, Flávia L. Adami, Ana I. S. Moretti, Vivian L. de Oliveira, Silvia B. Boscardin, Edecio Cunha-Neto, Jorge Kalil, Emmanuelle Jouanguy, Paul Bastard, Jean-Laurent Casanova, Mauricio Quiñones-Vega, Patricia Sosa-Acosta, Jéssica S. de Guedes, Natália P. de Almeida, Fábio C. S. Nogueira, Gilberto B. Domont, Keity S. Santos, Mayana Zatz

**Affiliations:** 1 Human Genome and Stem Cell Research Center, University of São Paulo, São Paulo, São Paulo, Brazil; 2 Department of Genetics and Evolutionary Biology, Biosciences Institute, University of São Paulo, São Paulo, São Paulo, Brazil; 3 Department of Pathology, School of Medicine, UNESP - São Paulo State University, Botucatu, São Paulo, Brazil; 4 Laboratório de Imunologia, Instituto do Coração (InCor), LIM19, Hospital das Clínicas da Faculdade de Medicina da Universidade de São Paulo, (HCFMUSP), São Paulo, Brazil; 5 Instituto de Investigação em Imunologia-Instituto Nacional de Ciências e Tecnologia-iii-INCT, São Paulo, Brazil; 6 Departamento de Clínica Médica, Disciplina de Imunologia Clínica e Alergia, Faculdade de Medicina da Universidade de São Paulo, São Paulo, Brazil; 7 Laboratory of Antigen Targeting to Dendritic Cells, Department of Parasitology, Institute of Biomedical Sciences, University of São Paulo, São Paulo, Brazil; 8 Laboratory of Human Genetics of Infectious Diseases, Necker Branch, INSERM U1163, Necker Hospital for Sick Children, Paris, France; 9 Imagine Institute, University of Paris, Paris, France; 10 St. Giles Laboratory of Human Genetics of Infectious Diseases, Rockefeller Branch, The Rockefeller University, New York, NY USA; 11 Proteomics Unit, Department of Biochemistry, Institute of Chemistry, Federal University of Rio de Janeiro, Rio de Janeiro, Brazil; 12 Laboratory of Proteomics (LabProt), Institute of Chemistry, LADETEC, Federal University of Rio de Janeiro, Rio de Janeiro, Brazil

**Keywords:** Covid-19, Supercentenarians, SARS-CoV-2, Elderly

## Abstract

**Background:**

Although older adults are at a high risk of severe or critical Covid-19, there are many cases of unvaccinated centenarians who had a silent infection or recovered from mild or moderate Covid-19. We studied three Brazilian supercentenarians, older than 110 years, who survived Covid-19 in 2020 before being vaccinated.

**Results:**

Despite their advanced age, humoral immune response analysis showed that these individuals displayed robust levels of IgG and neutralizing antibodies (NAbs) against SARS-CoV-2. Enrichment of plasma proteins and metabolites related to innate immune response and host defense was also observed. None presented autoantibodies (auto-Abs) to type I interferon (IFN). Furthermore, these supercentenarians do not carry rare variants in genes underlying the known inborn errors of immunity, including particular inborn errors of type I IFN.

**Conclusion:**

These observations suggest that their Covid-19 resilience might be a combination of their genetic background and their innate and adaptive immunity.

**Supplementary Information:**

The online version contains supplementary material available at 10.1186/s12979-022-00310-y.

## Background

The emergence of the Covid-19 pandemic resulted in more than six million deaths worldwide, with a higher risk for older adults and people with comorbidities to develop severe cases of the disease [1–4]. Covid-19 deaths of individuals over 60 represented over 70% of total Covid-19-related deaths in Brazil [5]. In the United States, about 80% of Covid-19 deaths have been among people older than 65 years [6]. The risk of dying from Covid-19 for an individual aged 85 years or more is 340 higher than for young adults (< 30 years old) [7].

One of the leading hypotheses for the higher Covid-19 severity in older people is a decrease in the immune response that occurs with aging [8]. The immunosenescence phenomenon is associated with significant changes in cytokine patterns and activation of inflammatory pathways, which result in the dysfunction of innate and adaptive immune responses [9, 10]. The immune cells’ senescence significantly contributes to immunity decline [11]. The thymus degenerates gradually with aging, resulting in a significant loss of diversity of the T cell repertoire, depletion, and/or diminished function of mature lymphocytes in secondary lymphoid tissues [12, 13]. Such a decline in immunity is responsible for higher susceptibility to infectious diseases and a decrease in the effectiveness of vaccinations in elderly cohorts [14, 15].

The chronic physiological stimulation of the immune system during life can establish the inflammaging phenomenon, characterized by a progressive and continuous increase of circulating levels of pro-inflammatory mediators [16, 17]. This pro-inflammatory basal state in the elderly may enhance the release of a large amount of pro-inflammatory cytokines as a response to the SARS-CoV-2 infection, which is directly correlated with lung tissue injury, multi-organ failure, and increased risk of dying from Covid-19 – the cytokine storm phenomenon [10, 18–20]. Also, comorbidities in older individuals, as a consequence of the multiple phenomena associated with organic aging [8], are strongly associated with an increased risk of Covid-19 complications, including sepsis and multiple organ dysfunction [21, 22].

In addition, recent studies reported the presence of pre-existing autoantibodies (auto-Abs) neutralizing type I IFNs in patients with life-threatening Covid-19 pneumonia, which block the antiviral activity of correspondent type I IFNs against SARS-CoV-2 [23]. As these auto-Abs skyrocket after age 65, they may represent an additional risk factor for critical Covid-19, especially in the elderly [24].

Despite that, worldwide reports of unvaccinated centenarians and supercentenarians (105 years or older) recovered from Covid-19 with mild or moderate symptoms called our attention [25–29]. Understanding why some individuals overcome the disease despite aging, such as the nun Ms. Randon, the oldest person already reported to survive Covid-19 at age 116 [30] is of great interest.

Host genotype influences how an individual responds to viral infections. For example, natural resistance to HIV-1 infection has been associated with a specific mutation in the *CCR5* gene [31]. In contrast, AIDS progression and many other infectious diseases are associated with specific alleles from the MHC, particularly *HLA-B* [32, 33]. In the context of Covid-19, the resistance to infection is still being investigated [34]. On the other hand, it has been reported that some genetic variants account for the variability in individuals’ susceptibility to Covid-19 and the severity of the disease. In this sense, a set of genes might explain how supercentenarians (some with comorbidities) overcome the disease without major complications.

Here, we present three cases of Brazilian supercentenarians who recovered from Covid-19 before the vaccination onset, including a 114 years old woman, the second oldest person in the world in this condition. Covid-19 in these three volunteers occurred in 2020 before new SARS-CoV-2 variants were reported in Brazil (especially Gamma variant - P.1). Aiming to enhance our comprehension of the underlying factors contributing to their resistance to the disease, we performed a comprehensive immunogenetic assessment and whole-exome sequencing.

## Results

### Humoral response against SARS-CoV-2

**Fig. 1:**
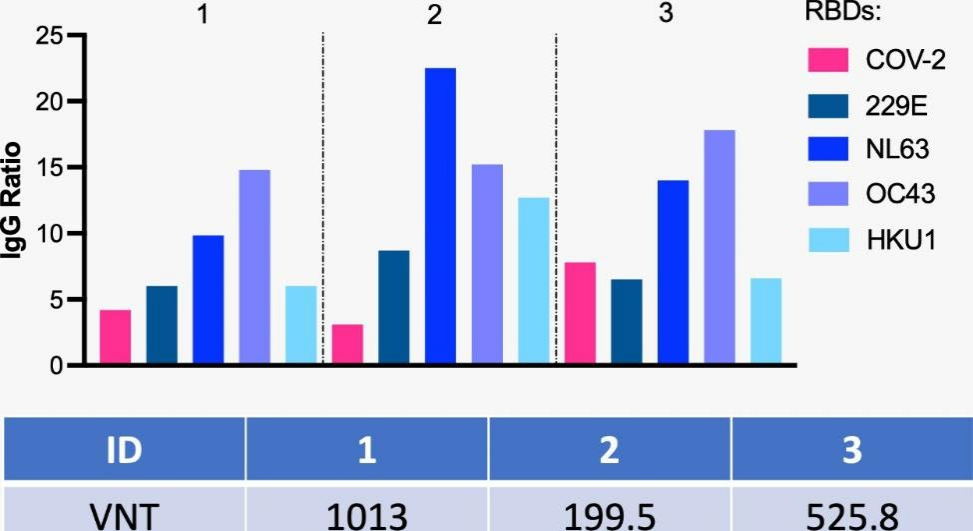
Supercentenarians present higher titers of IgG to RBDs of seasonal coronaviruses than for SARS-CoV-2 and neutralization titers above 160 (the minimum titer initially established by the FDA for convalescent plasma donors) [35]. The numbers represent the 3 individuals. The graph on top indicates IgG ratios for each RBD and the table above shows VNT for SARS-COV-2. VNT - Virus Neutralization Titers. Antibody levels expressed in ratios are shown for SARS-CoV-2 and the four seasonal coronaviruses. Neutralization titers are expressed in dilution. ID 02 was asymptomatic, and IDs 1 and 3 were symptomatic and recovered from Covid-19

Serological assays for SARS-CoV-2 RBD IgA, IgG, and IgM were performed through enzyme-linked immunosorbent assay (ELISA) for the Receptor-binding domain (RBD) of the Spike protein, and Nucleocapsid (NP) protein, at least four weeks after Covid-19 initial diagnosis. IgG seroconversion was detected for RBD and NP for all three individuals (Table 1). We must highlight that we performed these immune assays before the volunteers’ vaccination against Covid-19. Neutralization capacity was evaluated, and the asymptomatic individual produced low titers compared to the two who presented moderate symptoms. However, all of them presented titers above 160 (Fig. 1).

**Table 1:** Humoral immune-response profile of the presence of binding antibodies and type I autoantibodies of the volunteers.

BINDING ANTIBODIES AGAINST SARS-CoV-2*					
**Participants**			**ID 01**	**ID 02**	**ID 03**
**Covid-19 episode**			Symptomatic	Asymptomatic	Symptomatic
**Specific SARS-CoV-2antibodies** (Ratio)	**IgA**	**NP**	- (0.0)	- (0.2)	- (0.9)
		**RBD**	- (0.1)	- (0.5)	+ **(5.4)**
	**IgM**	**NP**	- (0.3)	- (0.0)	- (0.1)
		**RBD**	- (0.5)	+ **(1.5)**	- (0.7)
	**IgG**	**NP**	+ **(1.3)**	+ **(3.1)**	+ **(9.5)**
		**RBD**	+ **(4.2)**	+ **(3.1)**	+ **(7.8)**
**TYPE I IFN AUTOANTIBODIES**					
**Auto-anti IFNS IFN-α2, IFN-β and/or -ω** (Ratio)			- (0.0)	- (0.0)	- (0.0)

We also assessed pre-existing antibodies to RBD of the four common seasonal human coronaviruses (HCoV) α-CoV 229E, α-CoV NL63, β-CoV OC43, and β-CoV HKU1. All supercentenarians displayed IgG antibodies for all four HCoV tested, presenting higher titers for NL63 and OC43 (Fig. 1). Interestingly, IgG normalized values were generally lower for SARS-CoV-2 compared to HCoVs. IgG for SARS-COV-2 RBD did not correlate with neutralization titers among them, meaning that the one who presented the highest titer of RBD was not the one with the highest virus neutralization titer (Fig. 1).

Finally, autoantibodies (auto-Abs) neutralizing type I IFNs assays showed that none of the three supercentenarians had IFN-α, IFN-β, and/or INF-ω auto-Abs, based on negative results of luciferase-based immunoprecipitation (LIPS) assay. Table 1 summarizes the humoral responses assessed, except IgG for RBDs, shown in Fig. 1.

### Proteomic and metabolomic plasma analyses

**Fig. 2:**
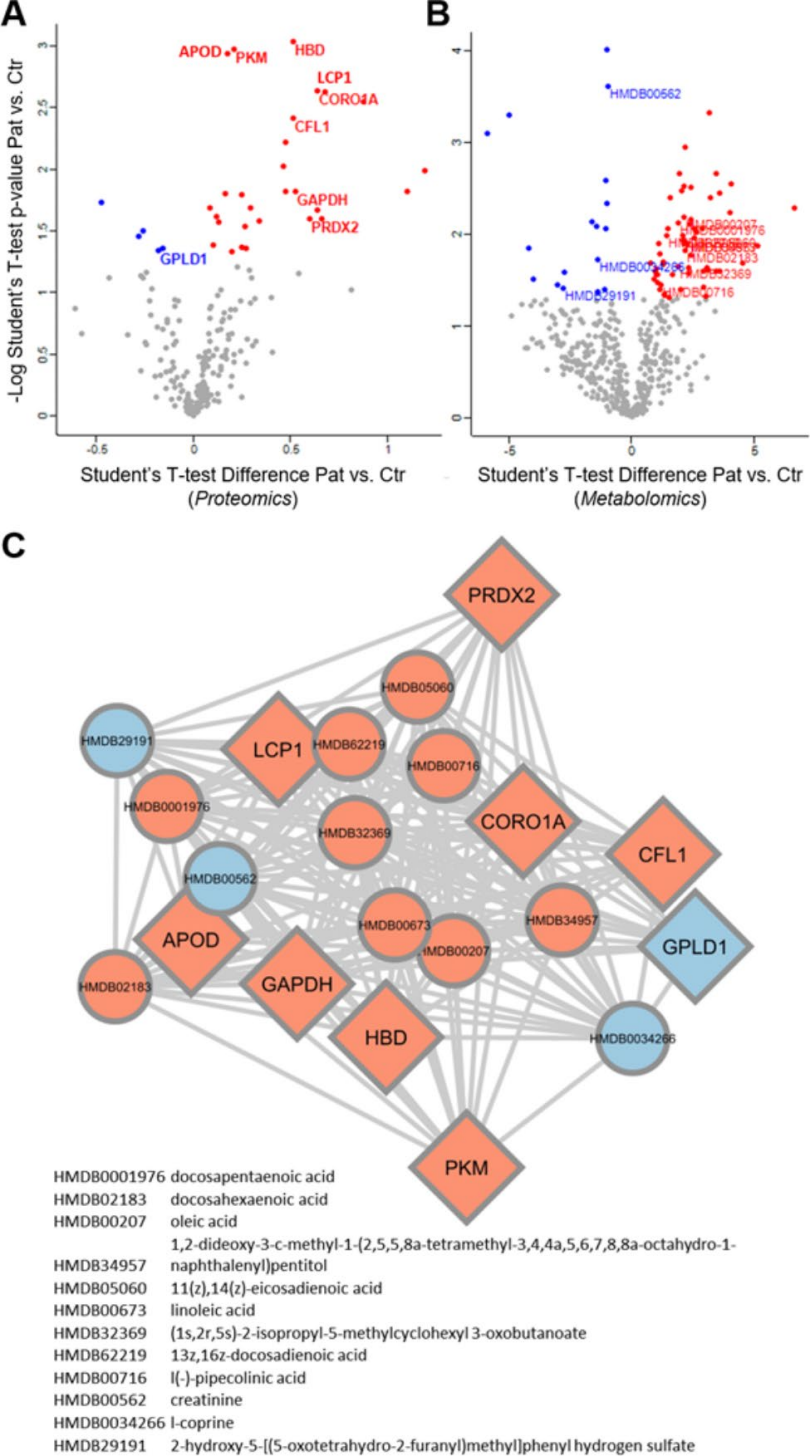
Plasma proteomics and metabolomics analyses of the supercentenarians infected with SARS-CoV-2 compared to healthy elderly subjects (> 95 years-old / non-infected). Volcano plots showing differentially expressed proteins **(A)** and metabolites **(B)**. Red and blue dots represent the up- and down-regulated proteins/metabolites, respectively. Protein-metabolite interaction network was built with the statistically significant proteins with OmicsAnalyst platform **(C)**. Red and blue nodes denote the up- and down-regulated proteins/metabolites, respectively. Circle and diamond node shapes represent metabolites and proteins, respectively

For label-free quantitative proteomics analysis, we compared the 3 supercentenarian’s plasma samples with 3 healthy subjects older than 95 years-old (non-infected). A total of 702 proteins were identified among all the samples analyzed. We performed a t-test to obtain the differentially expressed proteins between these two groups (Fig. 2 A). We found 33 altered proteins, 5 of them in low abundance (IGKV1-6, IGKV2-24, IGKV2-28, GPLD1, IGHV3-49) and 28 were up-regulated. The biological annotation enrichment of proteins which were up-abundant in the 3 supercentenarians showed processes associated with glycolytic pathways (P‑Value = 3.27E-05) and innate immune response like platelet aggregation (P‑Value = 3.75E-05), defense response to fungus (P‑Value = 1.81E-07), antimicrobial response (P‑Value = 5.21E-04).

The untargeted metabolomics approach detected 474 metabolites through positive (291 metabolites) and negative (183 metabolites) ionization modes. The proteomics statistical analysis was also applied to metabolomics (Fig. 2B). In the supercentenarians, 62 metabolites were up-regulated; while 17 metabolites were down-regulated. Enrichment analysis was mainly focused on the up-regulated metabolites, with the lower p-value for the biosynthesis of unsaturated fatty acids (P‑Value = 5.96E-4). Other metabolic pathways such as linoleic acid, purine, and ether lipid metabolisms were also enriched. Down-regulated metabolites presented an enrichment for the primary bile acid biosynthesis pathway. The protein-metabolite network represents the omics dataset’s integration (Fig. 2 C), and their convergence in the main disrupted biological processes and metabolic pathways: glycolysis and the immune system.

### Genetic ancestry

The genetic ancestry estimations for these three supercentenarians are shown in Table 2, which corresponds to an average of African ancestry (all volunteers) or Native American (ID 1) much higher than observed in the admixed Brazilian population from the same city [36–38].

**Table 2:** Supercentenarians’ continental ancestry composition.

ID	Age	Sex	European	African	Native American	East Asian
**01**	114	Female	0.34	0.43	0.23	0.00
**02**	111	Male	0.61	0.34	0.05	0.00
**03**	110	Male	0.00	1.00	0.00	0.00
Brazilian average genetic ancestry(average ± standard error) (36)			0.73 ± 0.26	0.18 ± 0.21	0.07 ± 0.07	0.03 ± 0.16

### Inborn errors of type I IFN immunity (IEI) genes

None of the supercentenarians carry rare variants in genes associated with inborn errors of Toll-like receptor 3 (TLR3) and interferon regulatory factor 7 (IRF7) dependent type I IFN immunity, which underlease life-threatening Covid-19 pneumonia [39, 40]. Also, we did not detect any copy number variation (CNV) in IEI genes for the three supercentenarians.

### MHC genes

Due to its central role in the adaptive immune response, genes from the MHC, especially those in the Human leukocyte antigen (HLA) system, are likely candidates to influence infection outcomes. Some HLA alleles were previously associated with severe Covid-19. Here we described three centenarians that recovered from Covid-19, which is a rare condition. Therefore, the small sample size does not allow an in-depth analysis of associated polymorphism. Nevertheless, since these data might be important to understand the mechanisms underlying HLA associations, and other groups evaluating centenarians might be interested in this data, we report the HLA alleles observed for all these centenarians as supplementary data (**Table S1**).

## Discussion

Despite the higher mortality risk associated with aging, analysis of exceptionally resilient supercentenarians may help to elucidate possible resistance mechanisms against SARS-CoV-2 infection in such extreme age.

The serological results of the three supercentenarians showed that they achieved seroconversion of IgG with robust levels against NP and RBD viral proteins. Such observations support the role of the humoral response against SARS-CoV-2. Our data corroborate a study by Foley and Colleagues, which reported higher anti-spike IgG antibody titers in nonagenarians and centenarians exposed to SARS-CoV-2 in a long-term care home (n = 15) than in younger individuals living in the same environment [27].

Neutralization analysis revealed that the asymptomatic individual produced lower titers than the two symptomatic ones. At first glance, this seems contradictory, but only a few asymptomatic individuals produce detectable neutralization titers [41] even with a low viral load during infection. On the other hand, Covid-19 patients admitted to a hospital usually display higher neutralizing antibody levels than mild disease or asymptomatic cases [42]. All three centenarians presented titers higher than 160, which is considered the cutoff for high levels [35].

We also assessed pre-existing antibodies to RBD of the four common seasonal HCoVs α-CoV 229E, α-CoV NL63, β-CoV OC43, and β-CoV HKU1. All supercentenarians displayed IgG antibodies for all four HCoV tested, presenting higher titers for NL63 and OC43. Interestingly, normalized values were generally lower for SARS-CoV-2 compared to HCoV. IgG for SARS-COV-2 RBD did not correlate with neutralization titers among them. The one who presented the highest titer of RBD was not the one with the highest virus neutralization titer.

The literature on the influence of pre-existing humoral immunity to HCoVs in SARS-CoV-2 infection is still controversial. Some studies have pointed out that immunity to the HCoVs has a protective effect on Covid-19 [43]. In contrast, others have described that pre-existing HCoV antibodies may hinder effective immunity against SARS-CoV-2 [44]. In our cohort, the supercentenarians displayed seroreactivity against all four HCoVs, as expected for the elderly, with normalized values showing high titers for all four HCoV. Previous results from a cohort of almost 400 Covid-19-infected individuals showed that HCoV immunity might impact disease severity, and patients with high HCoV reactivity are less likely to require hospitalization [45]. People infected with HCoV viruses during their life (and thus imprinted with that set of antigens/epitopes) would be protected later in life against infections with a related virus, as shown for Influenza [46]. Therefore, these high titers for seasonal coronaviruses might have positively impacted specific responses for SARS-CoV-2.

It is very likely that supercentenarians have been exposed to various pathogens in their life even when children and acquired active immunity making them more prone to defeat SARS-CoV-2. In this sense, it is tempting to speculate that the 1918 H1N1 influenza virus immunity could confer some protection against SARS-CoV-2 infection [47]. The hypothesis is that elderly born before 1918 could have developed immune memory cells able to recognize epitopes antigenically related to the H1N1 virus that would persist even one century later [48]. Interestingly, plasma samples of elderly who survived the Spanish flu pandemic revealed that neutralizing antibodies to the strain 1918 H1N1 influenza derived from isolated B cells have lasted a lifetime [49]. Our volunteers were born before 1918 and there is a report in Brazilian local media that ID03, who has lived his whole life in a region that was the most affected by the 1918 influenza virus, was infected by the virus as a child. It is possible that IDs 01 and 02 might have also been exposed to the Spanish flu in their early life, since both lived in regions affected by the virus but official data are lacking [50, 51].

Besides, a specific and unique subset of CD4 T cells has cytotoxic features in supercentenarians. These cells were accumulated during life exposition to pathogens. They might be considered an adaptation to aging since the immune system needs extra support to eliminate abnormal and infected cells. Such observation corroborates the hypothesis that the original antigenic sin phenomenon [52] could play a significant role in the recovery of the three supercentenarians through a mechanism of immunological memory [53].

It has been reported that circulating auto-Abs neutralizing type I IFNs (IFN-α and/or -ω) were distinctly found in elderly patients with severe Covid-19 and rarely detected in asymptomatic, benign infectious, or healthy individuals. They account for about 20% of critical Covid-19 cases in people over the 80s and total fatal Covid-19 cases [23, 24]. Interestingly, none of the three supercentenarians had neutralized auto-Abs against the type I IFNs, suggesting that they were not at a greater risk for complications in Covid-19 despite their advanced age.

Regarding the plasma proteomic and metabolomic analyses, we were able to compare these 3 supercentenarians with a control group of 3 individuals with comparable age, albeit no older than 110-years, whose serology was negative for Covid-19. Proteomics analysis showed that glycolytic proteins were more abundant in the supercentenarians compared to the control group, which is associated with the infection phisiopatology [54–57]. Krishnan and colleagues used targeted proteomics as well as untargeted metabolomics approaches in plasma samples and cell-line models and discovered that glycolysis and glutaminolysis are essential for virus replication [58]. In addition, during Covid-19 and other viral infections [59–61], there is a reprogramming of the glucose metabolism that overexpress glycolitic enzymes as glyceraldehyde 3-phosphate dehydrogenase - GAPDH (enriched in Fig. 2 C) in non-immune and immune cells, specially the ones involved in innate immunity - which are activated [54, 62]. In parallel, we observed that some processes related to innate immunity were found up-regulated in the plasma of these supercentenarians, demonstrating that they also displayed a first-line of defense capable of effectively neutralizing the infection in addition to their robust adaptive immune responses.

On the other hand, the metabolomics approach showed some up-regulated metabolites in the supercentenarians’ plasma related to fatty acid metabolism, especially the biosynthesis of unsaturated fatty acids. These molecules have a central role in modulating the immune pathways and inflammatory responses [63]. Some studies have described the importance of highly unsaturated fatty acids controlling both inflammation and thrombosis caused by Covid-19 [64–66]. The unsaturated fatty acid also mediates protein complex formation in lipid rafts and thus modulates SARS-CoV-2 entry gateways [67].

The genetic analysis indicated that all these three supercentenarians do not present variants associated with inborn errors of type I IFN immunity (IEI) genes, which is not surprising considering their advanced age without associated diagnosis. Because of the small sample size, conducting an in-depth analysis of polymorphisms associated with their Covid-19 resistance phenotype is not feasible. Nevertheless, their DNA WES data is available to the community for joint efforts to detect variants related to Covid-19 resistance.

Furthermore, the HLA alleles that each of these centenarians carry were described, being likely candidates to influence infection outcomes [68–70] and longevity [71]. Many studies have reported potential HLA alleles implicated in response to SARS-CoV-2 infection [72], whether they were identified in a specific geographic region or globally. This influence is suggestively related to differential antigen presentation and interaction with the T cell receptor [73].

## Conclusion

In the present study, we investigated three Covid-19 recovered supercentenarians (older than 110-years-old) who displayed robust IgG levels and neutralization titers against SARS-CoV-2. An enrichment of plasma proteins and metabolites related to innate immune response and host defense was observed. Despite their advanced age, none of them had neutralized auto-Abs against the type I IFNs. Also, they do not carry variants associated with inborn errors of type I IFN immunity (IEI) genes. They belong to a selected group of individuals with a long lifetime of pathogens’ expositions, immunity training, and genetic factors that lead them to develop mild symptoms not only against Covid-19 but also for several other diseases. Understanding the underlying mechanisms may be important to protect us from future pandemics.

## Methods

### Participants’ recruitment and Sample Collection

Three Brazilian unvaccinated supercentenarians who recovered from Covid-19 were contacted by our Human Genome and Stem Cell Research Center (HUG-CELL) research group following their report in national media: a 114-years-old woman (ID 01) and two men aged 111 (ID 02) and 110 (ID 03) years-old, respectively, at blood collection time. To our knowledge, these were the longest-lived people who recovered from Covid-19 in South America before the vaccination started and new SARS-CoV-2 variants emerged. RT-PCR tests confirmed the previous diagnosis of Covid-19, and all relevant clinical data related to the disease episode and comorbidities were collected from clinical reports and interviews. Baseline characteristics of the three supercentenarians are shown in Table 3.

**Table 3:** Demographic and clinical data of the participants

GENERAL INFORMATION			
**ID**	01	02	03
**Sex**	F	M	M
**Age**	114	111	110
**Year of birth**	1906	1910	1911
**Life status**	Death on Feb 2021, as a consequence of urinary tract infection	Death in Nov 2021 from natural causes	Still alive
Comorbidities*	Dementia	Diabetes *mellitus*	Hypertension
**Covid-19-RELATED EVENTS**			
**SARS-CoV-2 exposure/ beginning of symptoms**	August 2020	June 2020	May 2020
Disease severity**	Moderate	Asymptomatic	Moderate
**Hospital admission and discharge**	Hospital admission on Aug 31, 2020 ICU admission necessary	Not applicable	Hospital admission in June 2020 ICU admission necessary Hospital discharge after 18 days
**Positive SARS-CoV-2 serology**	Sep 2020 MAGLUMI 2019 nCoV IgM and IgM reagents	Jan 2021 ELISA IgM and IgG anti-Spike and anti-NP (Table 1)	Jun 2021 ELISA IgM and IgG anti-Spike and anti-NP (Table 1)

*Medical conditions associated with a higher risk for severe Covid-19 [74]

**According to the WHO classification of Covid-19 [75]

We collected peripheral blood samples of the volunteers from 30 to 120 days after the reported viral infections. For DNA extraction, samples were taken in vacutainer tubes with ethylenediaminetetraacetic acid - EDTA (BD Biosciences, USA, Catalog #. 360,057). Plasma and Serum were obtained by centrifugation for 10 min at 2000 x g at room temperature within 30 min after venipuncture. Then, the supernatant was transferred in aliquots of 1.5 mL into cryovials (Corning®, USA, Catalog #. 430,487). Samples were transferred to a -80 °C freezer until the moment of use.

### Humoral immune response assessment

The humoral immune response was analyzed by ELISA for IgA, IgM, and IgG-binding antibodies against the receptor-binding domain of Spike protein and NP protein of SARS-CoV-2. Besides, RBDs from human seasonal coronaviruses (HCoV) HKU-1, OC43, NL63, and 229E were also tested. RBDs from HCoVs were expressed in HEK293T cells, which plasmids are described in [76]. ELISA was performed using 96-well high-binding half-area polystyrene plates coated overnight at 4^o^C with 4 µg/mL of SARS-CoV-2 RBD, 0.8 µg/mL of the RBD of HCoVs, and 2 µg/mL NP (Kindly provided by Dr. Ricardo Gazzinelli, UFMG). Volunteers’ plasma samples were incubated at 56 °C for 30 min, diluted at 1:100, and run-in triplicates in ELISA. Results were given as the ratio of participant sample/average of a set of 20 control pre-pandemic samples. An antibody ratio of ≥ 1.2 was considered positive.

The detection of auto-Abs neutralizing type I IFNS (IFN-α2, IFN-β, and/or -ω) in plasma samples of the supercentenarians was assessed by LIPS assay, as described in [23]. Briefly, HEK293 cells transfected with type I IFNS fused to firefly luciferase were lysate (with doses from 0.1 pg/mL to 10 ng/mL of IFN-α2, -β, and/or -ω) and incubated with 10% diluted plasma of the volunteers. The resulting complexes were conjugated with agarose beads to capture the immune complexes. Then, the luciferase substrate furimazine was added to the reaction and the luminescence intensity (LU) was proportional to the presence of anti-Abs.

Neutralization titers were measured in a pseudovirus assay adapted from [77], only changing transfection to use lipofectamine 2000 (Thermo).

### Proteomics and metabolomics analyses

Plasma proteomics and metabolomics analyses from the supercentenarians were performed using tandem mass spectrometry. Detailed protocols concerning both analyses and data processing are available in Supplementary Methods. Three individuals older than 95 years-old who were not infected by SARS-CoV-2 and displayed a negative COVID-19 serology were included as the control group.

### Genomic assays

Whole-exome sequencing (WES) was performed in peripheral blood DNA with the Illumina NovaSeq platform at HUG-CELL facilities. Sequencing data were analyzed following bwa-mem and GATK Best Practices workflow, quality control, and annotation were performed as previously described [36]. HLA genes were realigned and called using hla-mapper [78], and the pipeline was described elsewhere [79].

### Genetic ancestry inference

The inference of genetic ancestry was performed in ADMIXTURE v1.36 [80], in supervised analysis (k = 4), after filtering the markers for linkage disequilibrium (r2 = 0.1) using a 50Kb sliding window with 10 kb steps, totaling 53,987 SNPs. Samples from both the 1000 Genomes Project [81] and the HGDP-CEPH [82] with over 95% inferred ancestry in a given group were used as parent populations, totaling 602 Africans, 624 Europeans, 630 East Asians, and 118 Native Americans.

## Electronic supplementary material

Below is the link to the electronic supplementary material.

Supplementary Material 1

Supplementary Material 2

## Data Availability

The genomic datasets presented in this study are currently being deposited at the “European Genome-phenome Archive (EGA)”, under accession number EGAS00001006376, a permanent public repository, in compliance with “Immunity and Ageing” recommendations and its open data policies. We will provide the public datasets accession numbers prior to publishing the final version.

## Abbreviations

Auto-Abs: Autoantibodies
CNV: Copy number variation
EDTA: Ethylenediamine tetraacetic acid
ELISA: Enzyme-linked immunosorbent assay
HCoV: Human coronaviruses
HLA: Human leukocyte antigen
HUG-CELL: Human Genome and Stem Cell Research Center
IEI: Inborn errors of type I IFN immunity
IFN: Interferon
IRF7: Interferon regulatory factor 7
LIPS: Luciferase-based immunoprecipitation
LU: Luminescence intensity
MHC: Major Histocompatibility Complex
NAbs: Neutralizing antibodies
NP: Nucleocapsid
RBD: Receptor-binding domain
TLR3: Toll-like receptor 3
WES: Whole-exome sequencing
