# Supplementary material for: The oldest unvaccinated Covid-19 survivors in South America
Source: Immun Ageing. 2022 Nov 16;19:57. doi: 10.1186/s12979-022-00310-y (PMC9666972; doi:10.1186/s12979-022-00310-y)
Supplement: Supplementary file 1 — Supplementary Material 1 [file 12979_2022_310_MOESM1_ESM.docx]

**he oldest unvaccinated Covid-19 survivors in South America**

Mateus V de Castro^1+^, Monize V. R. Silva^1+^, Michel S. Naslavsky^1,2^, Marilia O. Scliar^1,2^, Kelly Nunes^1,2^, Maria Rita Passos-Bueno^1,2^, Erick C. Castelli^3+^, Jhosiene Y. Magawa^4,5,6^, Flávia L. Adami^7^, Ana I. S. Moretti^4^, Vivian L. de Oliveira^4^, Silvia B. Boscardin^7^, Edecio Cunha-Neto^5,6^, Jorge Kalil^4,5^, [Emmanuelle Jouanguy](https://www.science.org/doi/10.1126/science.abd4585)^8,9^, Paul Bastard^8,9^, Jean-Laurent Casanova^9,10^, Mauricio Quiñones-Vega^11,12^, Patricia Sosa-Acosta^11,12^, Jéssica de Siqueira Guedes^11,12^, Natália Pinto de Almeida^11,12^, Fábio César Sousa Nogueira^11,12^, Gilberto Barbosa Domont^11^, Keity S. Santos^4,5,6+^, Mayana Zatz^1,2^*

+ Contributed equally to the manuscript

* Corresponding Author: Mayana Zatz ([mayazatz@usp.br](mailto:mayazatz@usp.br), phone 55 11 999817930);

**Institutions:**

^1^ Human Genome and Stem Cell Research Center, University of São Paulo, São Paulo, São Paulo, Brazil;

^2^ Department of Genetics and Evolutionary Biology, Biosciences Institute, University of São Paulo, São Paulo, São Paulo, Brazil;

^3^ Department of Pathology, School of Medicine, UNESP - São Paulo State University, Botucatu, São Paulo, Brazil;

^4^ Laboratório de Imunologia, Instituto do Coração (InCor), LIM19, Hospital das Clínicas da Faculdade de Medicina da Universidade de São Paulo, (HCFMUSP), São Paulo, Brazil;

^5^ Instituto de Investigação em Imunologia - Instituto Nacional de Ciências e Tecnologia-iii-INCT, São Paulo, Brazil;

^6^ Departamento de Clínica Médica, Disciplina de Imunologia Clínica e Alergia, Faculdade de Medicina da Universidade de São Paulo, São Paulo, Brazil;

^7^ Laboratory of Antigen Targeting to Dendritic Cells, Department of Parasitology, Institute of Biomedical Sciences, University of São Paulo, São Paulo, Brazil;

^8^ Laboratory of Human Genetics of Infectious Diseases, Necker Branch, INSERM U1163, Necker Hospital for Sick Children, Paris, France. University of Paris, Imagine Institute, Paris, France;

^9^ Imagine Institute, University of Paris, Paris, France;

^10^ St. Giles Laboratory of Human Genetics of Infectious Diseases, Rockefeller Branch, The Rockefeller University, New York, NY, United States;

^11^ Proteomics Unit, Department of Biochemistry, Institute of Chemistry, Federal University of Rio de Janeiro, Rio de Janeiro, Brazil;

^12^ Laboratory of Proteomics (LabProt), LADETEC, Institute of Chemistry, Federal University of Rio de Janeiro, Rio de Janeiro, Brazil.

**Supplementary Methods**

**Proteomic and metabolomic plasma analyses**

**Protein digestion**

Protein samples (100 µg) were diluted 20-fold with 5% SDS/50mM TEAB pH 8.5, reduced with DTT for 1h at 30°C and alkylated with 40mM IAA for 45 minutes at room temperature and protected from light. After acidifying with phosphoric acid to a final concentration of 1.2%, 7 volumes of 90% methanol/100mM TEAB (binding buffer) were added. Then the samples were placed on a S-Trap^TM^ MIDI spin columns (Protifi, USA) and centrifuged at 4000 g for 30 seconds and 9 volumes of binding buffer were added 3 times to remove detergent. Trypsin (Promega) digestion was done at a ratio of 1:50 (μg trypsin: μg protein) in a final volume of 350 µL of 50 mM TEAB buffer for 18 hours. The peptides were eluted in 3 steps by adding 3 solutions (250 µL of 50mM TEAB, 500 µL of 0.1% AF and 500 µL of 50% ACN/0.1% AF) followed by centrifugation at 4000 g for 60 seconds, dried using a speed-vac (SAVANT, Thermo Fisher Scientific), and stored at -30 °C.

**Metabolite extraction**

Plasma samples (75 µL) were extracted in ice-cold methanol (450 µL) enriched with an isotopic labeled internal standard (Testosterone-D3, 50 nM, LGC Standards; London, England). The samples were vortexed for 30 s and kept at -30 °C for 30 minutes. Then, were centrifuged at 14000g for 15 min at 4 °C, and the supernatants collected, dried, and reconstituted in 150 µL of 0.1% formic acid. A pool of all serum samples was used as quality control (QC). The metabolite extraction of QC was prepared following the same procedure used for the experimental samples. Blank samples were prepared without the biological matrix (i.e., serum plasma) to analyze possible contaminants and background signals.

**Proteomics analysis by tandem mass spectrometry**

Tryptic peptide mixtures were resuspended in 0.1% formic acids and quantified by the Qubit fluorometric assay according to the manufacturer´s instructions. Then, peptides were diluted to a final concentration of 0.5 µg/µL, and 2 µg of peptides were applied in duplicate in an Easy-nLC 1000 system (Thermo Fisher Scientific) coupled online to a Q-Exactive Plus mass spectrometer (Thermo Fisher Scientific). The peptides were loaded into a 3cm (length) x 100μm (internal diameter) pre-column, packed with 5 μm ReproSil-Pur C18 resin (Dr. Maisch GmbH), and then fractionated in an EASY-Spray column, 50 cm x 75 µm ID, PepMap RSLC C18, 2 µm (Thermo Fisher Scientific). The mobile phases were 5% ACN/0.1% formic acid (solvent A) and 95% ACN/0.1% formic acid (solvent B). The peptide mixture was separated with the gradient: 5-40% B for 167 min, 40-95% B for 5 min and 95% B for 8 min at 300 nL/min flow and 60 °C temperature. Full MS/DD-MS^2^ method follows full scan analysis (MS^1^), 70,000 resolution (m/z 200), AGC (Automatic Gain Control) of 3E6 and maximum injection time of 100 ms, and was configured to select the 20 most intense peptides for the fragmentation by High Energy Collision Dissociation (HCD), using a collision energy of 30% (NCE), 17,500 resolution (m/z 200), AGC of 1E6, maximum injection time of 50 ms, an isolation window of 1.4 m/z, and dynamic exclusion time of 40s.

**Metabolomics analysis by tandem mass spectrometry**

Untargeted metabolomics analysis was carried out in a Dionex Ultimate 3000 coupled to Q-Exactive Plus (Thermo Scientific, USA). The analysis was performed in a C18 column (Zorbax, 50 × 2.1 mm, 1.8 μm, Agilent, USA) using a mobile phase constituted of aqueous solution A and organic solution B. The column was held at 40 ºC, and the autosampler unit was maintained at 7 ºC. A volume of 10 µL of each sample was injected and analyzed in positive and negative modes. For analysis in positive mode, solutions A and B were respectively constituted by 0.1% formic acid and methanol acidified with 0.1% formic acid. On the other hand, for analysis in negative mode, solutions A and B were composed of 5mM ammonium formate (pH 8.0) and methanol, respectively. A gradient method (300 μL.min-1) was applied over 20 min, as follows: 5-10% B for 30 sec, 10-25% B for 1 min, 25-90% B for 8 min, 90-100% B for 4 min, and 100% B for 2 min.

The mass spectrometry analyses were performed using positive and negative electrospray ionization and scanning in full MS mode (m/z 70-1050) with the data-dependent acquisition (dd-MS2, top-10 DDA). The MS1 and MS2 parameters were set as follows: a) MS1 = resolution of 70 000, maximum IT 100 ms, AGC target 1E6; b) MS2 = resolution of 17 500, maximum IT 50 ms, AGC target 1E5, isolation window 2.0 m/z, loop count 10, HCD normalized collision energy of 30. The source ionization parameters were: spray voltage of 3.90 and 2.90 kV for positive and negative polarities, respectively; 380 °C capillary temperature; 380 °C auxiliary gas temperature; 20 auxiliary gas and 60 sheath gas.

Blank samples and quality controls (QC) were analyzed five different times in the same way as the experimental samples to assess the equipment's stability and reproducibility. Furthermore, the internal standard's peak shape, intensity, and retention time were monitored to evaluate the system's suitability.

**Proteomics data processing and statistical analysis**

FragPipe software 17.1 (https://fragpipe.nesvilab.org/) was used to process the raw data. Protein identification and validation were done with MSFragger 3.4 [(1)](https://www.zotero.org/google-docs/?lFxTRG) and Philosopher 4.1.1 [(2)](https://www.zotero.org/google-docs/?QQIbbc) algorithm versions. A human UniProt database was used for search (i.e., reviewed database with canonical proteins and isoforms, December 2021). MSFragger search parameters were semi-tryptic peptides with a maximum of two missed cleavage; as fixed modification was selected carbamidomethylation (C) and as variable modification were set oxidation (M) and acetylation (protein N-terminal); a precursor mass tolerance of 10 ppm and 0.1 Da of ion fragment mass tolerance. The False Discovery Rate (FDR) was < 1% at the peptide, PSM, and protein levels using the Percolator algorithm. Proteins were grouped into protein groups using ProteinProphet algorithm. Label-free quantification (LFQ) was done with the IonQuant [(3)](https://www.zotero.org/google-docs/?dktLRF) algorithm and the MaxLFQ method, using Match Between Runs (MBR). For LFQ, it was used the area of the two most abundant unique and razor peptides.

The list of identified proteins was imported to Perseus software (version 1.6.15.0) for data analysis. Only proteins detected in at least 70% of the samples were used for the statistical analysis. The average of the protein abundance was converted to log2 and normalized by subtracting the median of the sample distribution. The results obtained were analyzed using DAVID bioinformatic resource (Available at: https://david.ncifcrf.gov/).

**Metabolomics data processing and statistical analysis**

The deconvolution analyses of metabolomics data were carried out using Compound Discoverer 3.3 software (Thermo Fisher, USA) and an untargeted metabolomics workflow that matches and compares accurate mass and tandem mass experimental data with information from the following database: MZcloud, Human Metabolome Database (HMDB), Kyoto Encyclopedia of Genes and Genomes (KEGG), Lipid Maps, and BioCyc Database Collection (BioCyc). Five ppm of mass accuracy error and 1.2 s of retention time variance were accepted for metabolites detections. The identification of the metabolites was based on a spectrum-structure match of MS and MS/MS data of external libraries, providing a level 2 identification [(4)](https://www.zotero.org/google-docs/?n7vxZk)^]^.

For statistical analysis, the data were first normalized by log2 transformation and sample median subtraction. Univariate analysis was performed using Perseus software (version 1.6.15.0). Student T-test was used to identify the metabolites with different abundance (p-value < 0.05) between Patient vs. Control. Fold change analyses were also performed to identify up- and down-regulated metabolites. MetaboAnalyst 5.0 [(5,6)](https://www.zotero.org/google-docs/?UrKA0X) was employed to analyze the pathways associated with the dysregulated metabolites.

**Protein-metabolite network**

OmicAnalyst [(7)](https://www.zotero.org/google-docs/?ZOPTdO) platform was employed to build the protein-metabolite interaction network. Re-normalization was required after data filtering, employing for both datasets a normalization by sum and pareto scaling. DIABLO (Data Integration Analysis for Biomarker discovery using Latent cOmponents) method was chosen to create the correlation network with a correlation threshold (within-omics) of 0.8. Cytoscape software (version 3.9.1) was used to visualize and edit the network [(8)](https://www.zotero.org/google-docs/?H1STAY)^]^.

**References**

[1. Kong AT, Leprevost FV, Avtonomov DM, Mellacheruvu D, Nesvizhskii AI. MSFragger: ultrafast and comprehensive peptide identification in mass spectrometry–based proteomics. Nat Methods. 2017 May;14(5):513–20.](https://www.zotero.org/google-docs/?mnbBSo)

[2. da Veiga Leprevost F, Haynes SE, Avtonomov DM, Chang HY, Shanmugam AK, Mellacheruvu D, et al. Philosopher: a versatile toolkit for shotgun proteomics data analysis. Nat Methods. 2020 Sep;17(9):869–70.](https://www.zotero.org/google-docs/?mnbBSo)

[3. Yu F, Haynes SE, Nesvizhskii AI. IonQuant Enables Accurate and Sensitive Label-Free Quantification With FDR-Controlled Match-Between-Runs. Mol Cell Proteomics [Internet]. 2021 Jan 1 [cited 2022 Aug 18];20. Available from: https://www.mcponline.org/article/S1535-9476(21)00050-5/abstract](https://www.zotero.org/google-docs/?mnbBSo)

[4. Schymanski EL, Jeon J, Gulde R, Fenner K, Ruff M, Singer HP, et al. Identifying Small Molecules via High Resolution Mass Spectrometry: Communicating Confidence. Environ Sci Technol. 2014 Feb 18;48(4):2097–8.](https://www.zotero.org/google-docs/?mnbBSo)

[5. Pang Z, Chong J, Zhou G, de Lima Morais DA, Chang L, Barrette M, et al. MetaboAnalyst 5.0: narrowing the gap between raw spectra and functional insights. Nucleic Acids Res. 2021 Jul 2;49(W1):W388–96.](https://www.zotero.org/google-docs/?mnbBSo)

[6. Chong J, Soufan O, Li C, Caraus I, Li S, Bourque G, et al. MetaboAnalyst 4.0: towards more transparent and integrative metabolomics analysis. Nucleic Acids Res. 2018 Jul 2;46(W1):W486–94.](https://www.zotero.org/google-docs/?mnbBSo)

[7. Zhou G, Ewald J, Xia J. OmicsAnalyst: a comprehensive web-based platform for visual analytics of multi-omics data. Nucleic Acids Res. 2021 Jul 2;49(W1):W476–82.](https://www.zotero.org/google-docs/?mnbBSo)

[8. Shannon P, Markiel A, Ozier O, Baliga NS, Wang JT, Ramage D, et al. Cytoscape: a software environment for integrated models of biomolecular interaction networks. Genome Res. 2003 Nov;13(11):2498–504.](https://www.zotero.org/google-docs/?mnbBSo)
