# Supplementary material for: The oldest unvaccinated Covid-19 survivors in South America
Source: Immun Ageing. 2022 Nov 16;19:57. doi: 10.1186/s12979-022-00310-y (PMC9666972; doi:10.1186/s12979-022-00310-y)
Supplement: Supplementary file 2 — Supplementary Material 2 [file 12979_2022_310_MOESM2_ESM.docx]

**Table S1. HLA alleles in unvaccinated supercentenarians recovered from Covid-19**

| **ID** | **01** | | **02** | | **03** | |
| --- | --- | --- | --- | --- | --- | --- |
| **Gene** | **Allele 1** | **Allele 2** | **Allele 1** | **Allele 2** | **Allele 1** | **Allele 2** |
| **HLA-A** | *24:02 | *02:02 | *34:02 | *36:01 | *68:02 | *74:01 |
| **HLA-B** | *51:01 | *18:01 | *18:01 | *44:01 | *15:03 | *07:02 |
| **HLA-C** | *07:01 | *05:01 | *02:02 | *04:01 | *07:02 | *02:10 |
| **HLA-G** | *01:03 | *01:04 | *01:01 | *01:04 | *01:01 | *01:01 |
| **HLA-E** | *01:03 | *01:03 | *01:01 | *01:01 | *01:01 | *01:03 |
| **HLA-F** | *01:01 | *01:01 | *01:02 | *01:03 | *01:01 | *01:01 |
| **HLA-DRA** | *01:01 | *01:01 | *01:01 | *01:01 | *01:01 | *01:01 |
| **HLA-DRB1** | *07:01 | *13:01 | *07:01 | *15:03 | *15:03 | *15:03 |
| **HLA-DQA1** | *02:01 | *03:01 | - | - | *01:02 | *01:02 |
| **HLA-DQB1** | *02:02 | *06:03 | - | - | *06:02 | *06:02 |
| **MICA** | *001 | *009 | *001 | *004 | *008 | *008 |
| **MICB** | *002 | *008 | *005 | *008 | *005 | *008 |
| **HLA-DOB** | *01:01 | *01:03 | *01:01 | *01:03 | *01:01 | *01:01 |

- HLA-DQ typing failed for this sample
